# Blowfly puparia in a hermetic container: survival under decreasing oxygen conditions

**DOI:** 10.1007/s12024-017-9892-3

**Published:** 2017-07-01

**Authors:** Anna Mądra-Bielewicz, Katarzyna Frątczak-Łagiewska, Szymon Matuszewski

**Affiliations:** 1 Laboratory of Criminalistics, Adam Mickiewicz University, Św. Marcin 90, 61-809 Poznań, Poland; 2 Department of Animal Taxonomy and Ecology, Adam Mickiewicz University, Umultowska 89, 61-614 Poznań, Poland

**Keywords:** Forensic entomology, Post-mortem interval, Insect evidence, Preservation techniques, Hypoxia

## Abstract

Despite widely accepted standards for sampling and preservation of insect evidence, unrepresentative samples or improperly preserved evidence are encountered frequently in forensic investigations. Here, we report the results of laboratory studies on the survival of *Lucilia sericata* and *Calliphora vomitoria* (Diptera: Calliphoridae) intra-puparial forms in hermetic containers, which were stimulated by a recent case. It is demonstrated that the survival of blowfly intra-puparial forms inside airtight containers is dependent on container volume, number of puparia inside, and their age. The survival in both species was found to increase with an increase in the volume of air per 1 mg of puparium per day of development in a hermetic container. Below 0.05 ml of air, no insect survived, and above 0.2 ml of air per 1 mg of puparium per day, survival reached its maximum. These results suggest that blowflies reveal a single, general pattern of survival under decreasing oxygen conditions and that this pattern is a product of number of developing insects, their age and the initial amount of available air. Implications for forensic entomology are discussed.

## Introduction

The importance of insect evidence in criminal investigations has increased substantially over recent decades [1]. However, in practice it is extremely rare that a qualified forensic entomologist is present at the crime scene. Usually, insects are collected by crime scene technicians or medical examiners. Although there are widely accepted protocols for sampling and preservation of insect evidence [1–5], unrepresentative samples or improperly preserved evidence are encountered frequently in forensic investigations [1, 6].

The blowfly puparium is an opaque, barrel-like structure; a prepupa, a pupa or a pharate adult (i.e. intra-puparial forms) develop inside it [7, 8]. When puparia contain developing insects, it is recommended to preserve some of them for laboratory rearing and some as dead specimens [1, 5]. “Living” puparia should be kept on a damp soil (or a similar substrate) with constant air access and should be transferred within 24 h for rearing in laboratory conditions [1, 4, 5]. The other portion should be killed with boiling water and kept in 70%–95% ethanol in the fridge if possible, after piercing the puparium to allow the preservative to enter inside [9, 10]. Despite these standards, puparia are sometimes improperly preserved, for example, in a hermetic container with no preservative inside or in a leaking container resulting in evaporation of preservative and dehydration of specimens [6]. Incorrect preservation may affect the scope of laboratory procedures to be performed using intra-puparial forms. Moreover, in some instances it may elicit extra questions, which are irrelevant in cases with properly preserved insect samples.

Here we report results of experiments provoked by a recent case in which fly puparia were improperly preserved. The senior author received the insect evidence preserved in an airtight glass jar with no preservative inside. Almost four months passed between insect sampling at the crime scene and the arrival of specimens at the laboratory. Inspection revealed that the jar contained 14 adult insects (11 true flies, among them a single specimen of the flesh fly *Sarcophaga* sp., and three specimens of parasitoid wasp *Nasonia vitripennis* (Walker, 1836)), 18 closed puparia with decaying pupae inside, two puparia with small holes and decaying pupae inside, and a single empty puparium. All puparia belonged to *Sarcophaga argyrostoma* (Robineau-Desvoidy, 1830). The report from the crime scene indicated that crime technicians sampled 21 puparia and 10 adult flies, the latter only from the families Calliphoridae, Muscidae and Fannidae. Accordingly, a single specimen of adult *Sarcophaga* and three specimens of adult *N. vitripennis* must have emerged inside the jar. Because the oldest specimen was the puparium of *S. argyrostoma* from which the adult emerged in the jar, minimum post-mortem interval was inferred from the total immature development of *S. argyrostoma* minus the period of development in the jar. No scientific data on the survival of intra-puparial forms inside hermetic containers were available at the time of case analysis. Accordingly, the senior author estimated (based on jar volume and number of puparia inside) that insects might have been developing in the jar for no more than 5 days. However, the estimate was subjective and therefore prone to error. Consequently, it was decided to make a basic study to answer the following questions provoked by the circumstances of the case. How long may intra-puparial forms survive inside a hermetic container? Does the number of puparia in the container affect their survival? Does the age of insect inside the puparium affect its ability to survive inside the container under conditions of decreasing oxygen? Are forensically important species equally sensitive to hypoxia?

Living organisms can experience hypoxia at high altitudes [11], inside mammalian stomachs [12], in dung [13] or carrion [14], in hermetic containers [15], and in temporarily immersed substrates [11, 16]. In the case of holometabolic insects, the ability to survive in hypoxic conditions is often stage-specific [11]. Metabolically highly active stages, for example, pupae, are most sensitive to hypoxia. The survival rate of larvae and intra-puparial forms after submergence in water was studied for several forensically important blowflies: *Phormia regina* (Meigen, 1826) [17, 18], *Protophormia terraenovae* (Robineau-Desvoidy, 1830), *Calliphora vicina* Robineau-Desvoidy, 1830, *Cochliomyia macellaria* (Fabricius, 1775), *Lucilia sericata* (Meigen, 1826) [18], *Chrysomya albiceps* (Wiedemann, 1819), *C. megacephala* (Fabricius, 1794), and *C. putoria* (Wiedemann, 1830) [19]. These studies indicated that the survival is affected by the amount of time a life stage is submerged and its age at submergence [17, 18]. The survival of intra-puparial forms of blowflies was, however, not studied under decreasing oxygen conditions as experienced in airtight containers. Different conditions in hermetic containers, compared to underwater environments, may result in different survival rates of forensically important blowflies.

## Materials and methods

### General protocol

*Lucilia sericata* and *Calliphora vomitoria* (Linnaeus, 1758) were chosen for the experiments as they are frequently reported from forensic cases [20, 21] and pig carcass experiments in Central Europe [22–24]. Larvae were purchased from a fishing shop before each trial. Experiments were performed in insect incubators at 22.5 °C for a photoperiod (h) of 12:12 (L:D). Glass jars (twist type with a metal lid) were used as containers. Only fresh puparia (sampled shortly after puparial darkening had started, within 5 h from pupariation) were selected for the experiments. Upon termination of each trial, adult flies that emerged were counted. Tenerals, which died during emergence, were also counted. Closed puparia were left in open containers for an additional seven days to double-check the mortality.

### Experiment 1: factors affecting the survival of intra-puparial forms in hermetic containers

In order to choose factors of interest and test the protocol, several preliminary studies were conducted. The main experiment was performed according to a factorial block design. Three blocks (trials) were separated in time. Based on results of preliminary studies, it was decided to test effects of the following factors: blow fly species, number of puparia, container volume, damp paper substrate, and container conditions, with two levels each (Table 1). Puparia of both species were placed in each container (ratio 1:1) and containers were either left open or hermetically sealed (hermetic). Single fold paper towels (25 × 23 cm) were used as a paper substrate, hydrated with 3 ml of water at the onset of the study in sealed containers. In the case of open containers the same size of paper towels were used and they were hydrated with 3 ml of water once a day during the study. Each trial included 16 containers and was terminated after 14 days.

**Table 1:** Factors included in the experiments

Factor	Levels	E1	E2
Species	I. *Lucilia sericata*	+	+
	II. *Calliphora vomitoria*	+	+
Number of puparia	I. 10	+	+
	II. 20	−	+
	III. 30	+	+
Container volume	I. 315 ml	+	+
	II. 900 ml	+	−
Damp paper substrate	I. Present	+	−
	II. Absent	+	+
Container conditions	I. Open	+	−
	II. Hermetic	+	+
Age of intra-puparial forms	10 levels (from 3 days old until 7.5 days old)	−	+

E1, E2 - experiment 1 and 2

### Experiment 2: survival of intra-puparial forms of various ages in hermetic containers

This experiment included two factors: age of intra-puparial forms (10 levels) and number of puparia (three levels) (Table 1). A block design was used with three blocks (trials), separated in time. Containers of 315 ml were used with no paper inside. Puparia of both species (ratio 1:1) were put twice daily into the containers, which were then sealed, starting from 3- to 7.5 day-old insects. Each trial included 30 containers and was terminated after 14 days.

### Data analyzes

Results of experiment 1 were tested using factorial analysis of variance. After analyzing individual factors, the number of puparia and the container volume were transformed into air volume per puparial weight. For this purpose, the volume of air inside the container at the onset of the trial was divided by the total mass of puparia put into the container. Consequently, air volume per 1 mg of puparium was obtained. Average mass of a fresh puparium was used in these calculations (28.9 mg for *L. sericata* and 85.2 mg for *C. vomitoria*).

In experiment 2, the survival of intra-puparial forms was used as a response variable and air volume per 1 mg of puparium per day as a predictor variable. To obtain the predictor variable, air volume per 1 mg of puparium (see the previous section) was divided by a predicted period of development in the container. The predicted development in the container is the total intra-puparial development (10.3 days at 23 °C for *C. vomitoria* [25] and 6.2 days at 22 °C for *L. sericata* [26]) minus the age of intra-puparial forms at the time of placement in the container. The relationship was analyzed using nonlinear regression. The polynomial model (*y* = *b*_0_ + *b*_1_ *x* + *b*_2_ *x* ^2^) was fitted with the Levenberg–Marquardt procedure for parameters approximation.

In the analysis 5% level of significance was accepted. Calculations were made using Statistica 10 (StatSoft, Inc., 1984–2011).

## Results

### Factors affecting the survival of intra-puparial forms in hermetic containers

**Fig. 1:**
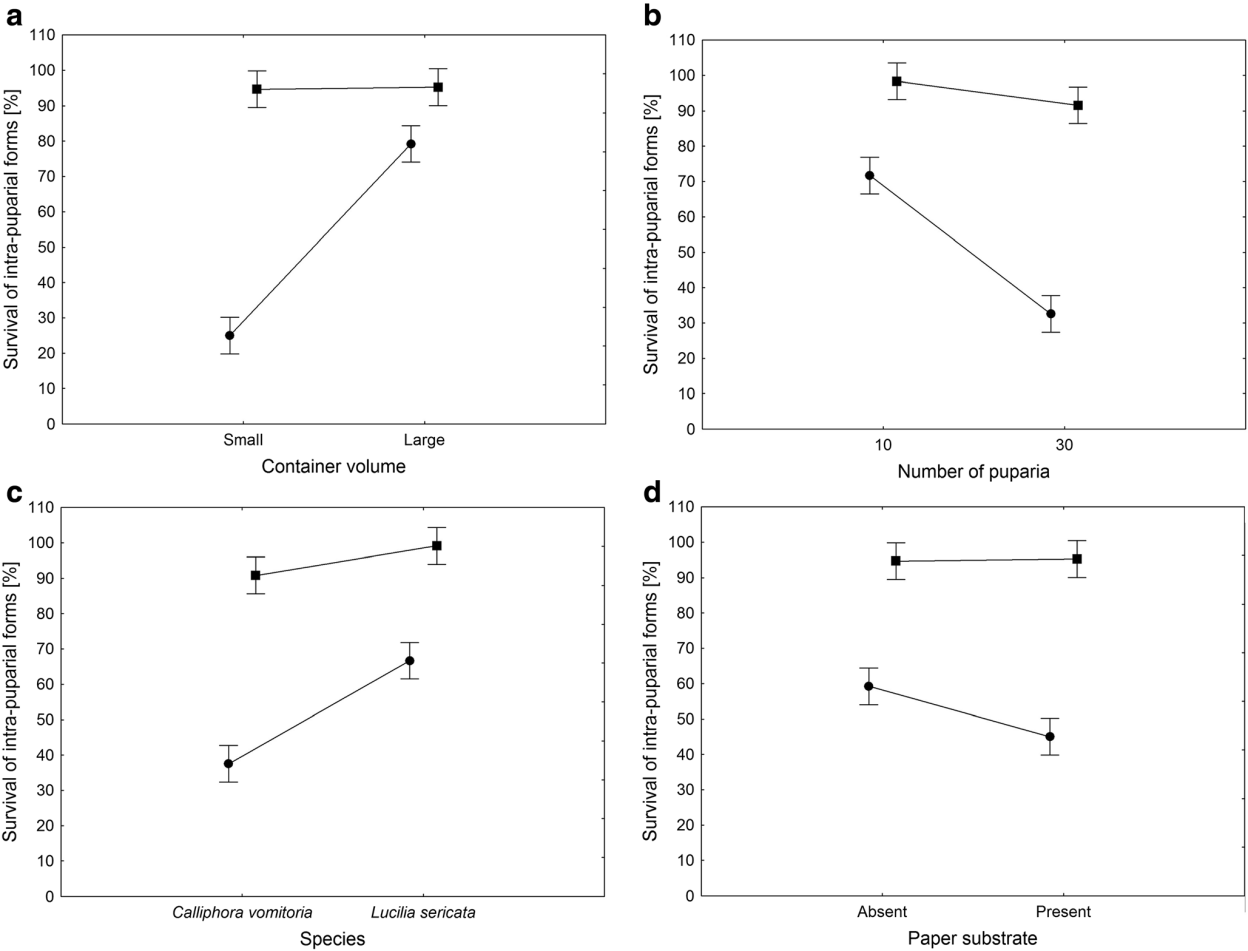
Effect of container volume (**a**), number of puparia inside the container (**b**), species (**c**) and presence of damp paper substrate inside the container (**d**) on mean survival of intra-puparial blowflies inside open (■) and hermetic containers (●). Vertical bars represent 95% confidence intervals. Data were pooled to show main effects

**Fig. 2:**
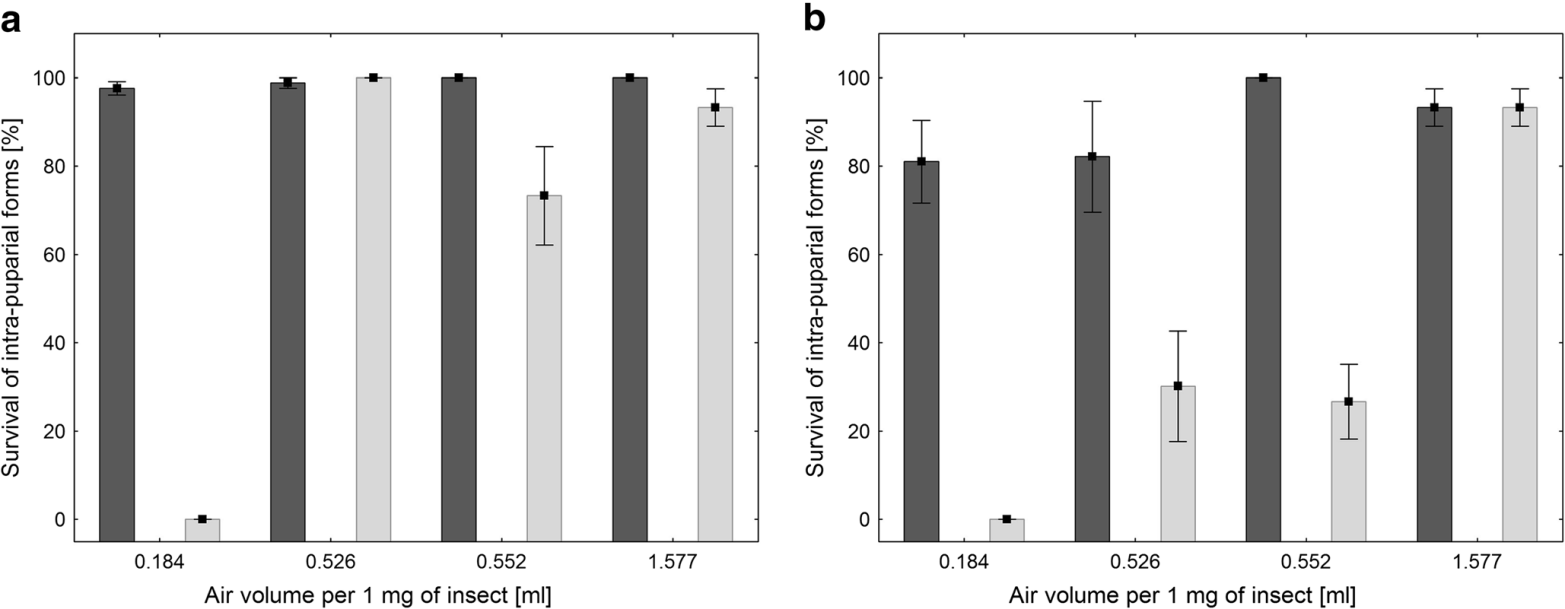
Mean (± SE) survival of *Lucilia sericata* (**a**) and *Calliphora vomitoria* (**b**) intra-puparial forms at different volume of air per puparial weight in open () and hermetic containers ()

Container conditions, container volume, species, number of puparia, and paper substrate significantly affected the survival of intra-puparial forms inside containers (Table 2). The survival rate was distinctly lower in hermetic containers, and this was the largest observed effect. Moreover, container conditions significantly interacted with all the other factors (Table 2). In all cases, the differences between treatments in hermetic containers were clearly larger than the differences in open containers (Fig. 1). Lower container volume and higher density of puparia resulted in a substantial decrease of survival (Fig. 1, Table 2). *Lucilia sericata* revealed higher survival rates than *C. vomitoria* (Fig. 1)*.* Damp paper reduced the survival rate in airtight containers (Fig. 1). Transforming the container volume and the number of puparia into air volume per puparial weight revealed that the survival of intra-puparial forms inside hermetic containers is positively related to the volume of air per 1 mg of puparium at the time of placement in the container (Fig. 2).

**Table 2:** Effects of experimental factors on the survival of intra-puparial forms

Factor/interaction	*F*	*P*	Partial *eta* ^*2*^
Container volume	111.8	<0.001	0.64
Damp paper substrate	6.9	0.011	0.10
Species	52.3	<0.001	0.45
Number of puparia	78.4	<0.001	0.55
Container conditions	273.6	<0.001	0.81
Container volume * Damp paper substrate	0.002	0.968	0.00
Container volume * Species	1.2	0.268	0.02
Damp paper substrate * Species	0.7	0.420	0.01
Container volume * Number of puparia	8.1	0.006	0.11
Damp paper substrate * Number of puparia	1.5	0.223	0.02
Species * Number of puparia	4.3	0.041	0.06
Container volume * Container conditions	107.1	<0.001	0.63
Damp paper substrate * Container conditions	8.1	0.006	0.11
Species * Container conditions	16.1	<0.001	0.20
Number of puparia * Container conditions	39.0	<0.001	0.38

### Survival of intra-puparial forms of various ages in hermetic containers

**Fig. 3:**
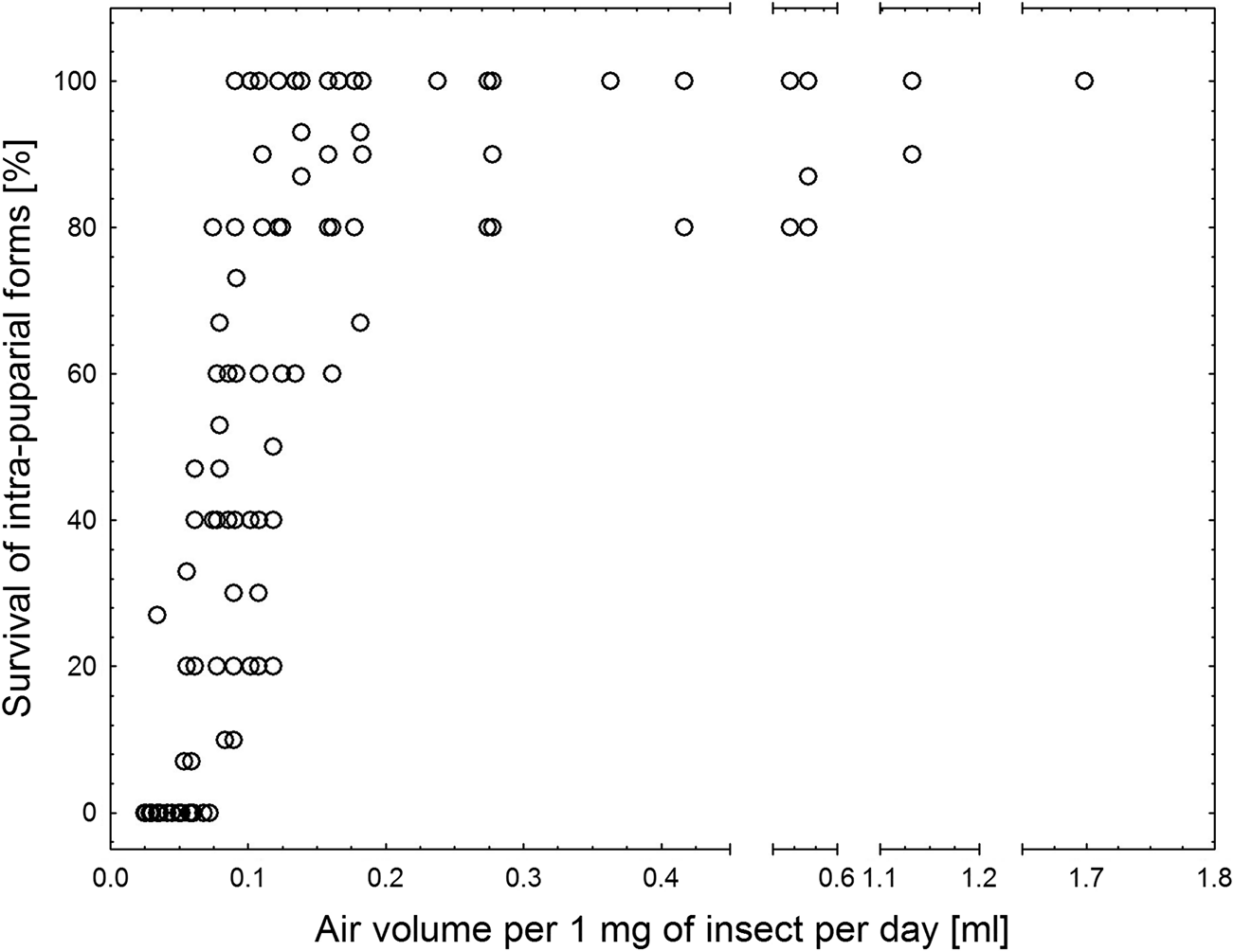
Survival of intra-puparial forms (pooled *Lucilia sericata* and *Calliphora vomitoria*) in hermetic containers plotted against air volume per 1 mg of puparial weight per day of intra-puparial development

**Fig. 4:**
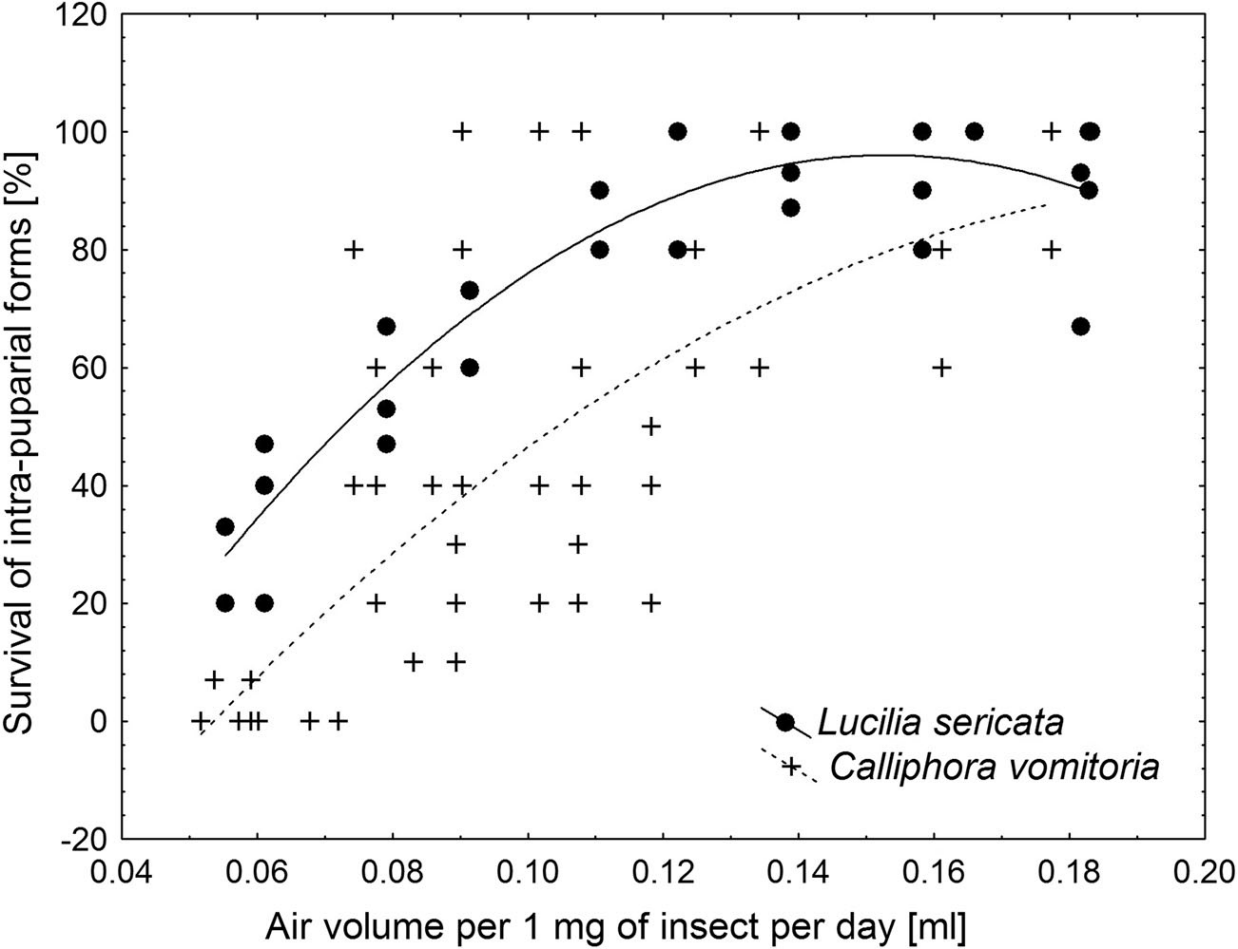
The polynomial models for the relationship between survival of intra-puparial forms (*Lucilia sericata* or *Calliphora vomitoria*) in hermetic containers and air volume per 1 mg of puparial weight per day inside container

Survival was found to increase with the initial age of an insect inside the puparium. It increased with increase in the volume of air per 1 mg of puparium per day of the intra-puparial development inside the container (Fig. 3). The pattern of changes was similar for both species, with polynomial increase of survival between 0.05 and 0.2 ml of air per 1 mg of puparium per day (*L. sericata*, *y* = (−70.41) + (2172.28) × *x* + (−7086.6) × *x* ^2^, *P* < 0.001, *r* ^*2*^ = 0.85; *C. vomitoria*, *y* = (−73.804) + (1580.01) × *x* + (−3772.7) × *x* ^2^, *P* < 0.01 for *b*_0_ and *b*_1_, *P* = 0.09 for *b*_2_, *r* ^*2*^ = 0.58; where *y* is the survival of intra-puparial forms and *x* is air volume per 1 mg of puparium per day; Fig. 4). Below 0.05 ml of air per 1 mg of puparium per day, no insect survived, and above 0.2 ml of air per 1 mg of puparium per day, the survival reached its maximum (Fig. 3).

## Discussion

This study has revealed that container volume, number of puparia inside, and their age are the factors of highest importance for the survival of blowfly intra-puparial forms inside hermetic containers. Experiments with submerged puparia indicated that survival is determined by the time of submergence and the age of the insect [18, 19]. Moreover, in submersion conditions, the pattern of survival was found to inversely track oxygen consumption during metamorphosis, with decrease of survival in the white puparial stage and pharate adult stage [18]. This study demonstrated that in hermetic containers survival increases with the increase of insect’s age at the moment of placement inside the container. These differences may be explained by different stress agents to which insects are exposed in hermetic containers and after submersion under water. In underwater conditions, oxygen deprivation seems to be the most important and the only permanent stress agent (water temperature or water contamination with toxic substances are not permanent stressors). However, in airtight containers, the set of permanent stress agents is more complex. Apart from gradual oxygen deprivation, gradual increase of carbon dioxide and other by-products of insect metabolism are permanent stressors [15]. Moreover, in such conditions, some insects will inevitably die, resulting in putrefaction of their bodies and resultant accumulation of volatile by-products of decay. All these accumulating substances are toxic for insects. Accordingly, their gradual increase in hermetic containers may be similarly or even more important for survival than gradual decrease of oxygen level. From this point of view, large effects of container volume and number of puparia inside, as revealed in this study, may simply result from the higher rate of toxins accumulation in small containers and at higher densities of puparia inside.

Intra-puparial *C. vomitoria* revealed lower survival rate in hermetic containers, compared to *L. sericata*, which is in line with the results of previous studies [18]. The intra-puparial development lasts longer in *C. vomitoria* than in *L. sericata* [25–28]. Therefore, in airtight containers, it can be assumed that puparia of *C. vomitoria* had not completed development by the time the atmosphere had become lethal, whereas *L. sericata*, due to its shorter intra-puparial development time, usually completed its development before the conditions in the container became lethal.

We believe that the synthetic measure developed during this study (i.e. the air volume per 1 mg of puparium per day of intra-puparial development) may be used to describe the survival of all forensically relevant intra-puparial forms inside hermetic containers. The survival patterns of *L. sericata* and *C. vomitoria* were surprisingly similar when plotted against this measure. This finding suggests that the survival of intra-puparial forms of blowflies in airtight containers may be described by a single general model. We think that the model given in this article (Figs. 3 and 4) reasonably approximates this general one. However, the question of whether the general “window for survival” lies between 0.05 and 0.2 ml of air (per mg of puparium per day) needs to be studied further with more species.

The measure discussed in the previous section has clear practical advantages. It may easily be calculated for any container and any number of puparia inside. Therefore, in cases as the one being an inspiration for this research, that is, when intra-puparial forms had died or adult flies had emerged inside the container before arrival to the laboratory, it may be used to roughly estimate the age of intra-puparial forms when they were placed into the container. Based on the number of adult insects, which has emerged in the container, the amount of air per puparial weight per day may be estimated using the models given in this article or by simply inferring the values from the graphs (Figs 3 and 4). Then, having the knowledge of the container volume and the number of puparia inside, one may infer the amount of time insects have been developing in the container. For example, if all puparia of *C. vomitoria* in the container gave adult flies (i.e. the survival was 100%), we may deduce from Figs. 3 and 4 that the amount of air per 1 mg of puparium per day must have been above 0.09 ml and most probably above 0.2 ml. If the container is 300 ml in volume and there are 10 puparia inside (in total 852 mg), the amount of air per 1 mg of puparium will be 0.352 ml. Keeping in mind the daily amounts of air, which provides 100% survival, we may calculate that at 0.09 ml of air per day, insects might have been developing in the container for no more than about 3.9 days, and at 0.2 ml of air per day, the development would occur for no more than about 1.8 days. The survival patterns presented in this article may also be useful to calculate the maximum possible development in the given container and at a given number of puparia inside. As no adult emerged below 0.05 ml of air per day, this value may be the lower limit for survival and it may be used to calculate the maximum possible development time in the container. If for example, 21 puparia of *S. argyrostoma* (about 3150 mg) were put into a 300 ml container, about 0.095 ml of air would be available per 1 mg of puparium inside the container. Using the lower limit for survival, we get 1.9 days of maximum possible development in the container. Accordingly, if the current results would have been available when the case had been analyzed and assuming that temperature conditions had been similar to the experimental temperature from this study (i.e. about 22.5 °C), the PMI estimate would be more accurate by about 3 days (the estimate changed from 5 days in the jar to 2 days in the jar).

Future studies should address the effect of temperature on the survival rate of blowflies under the decreasing oxygen conditions. Due to the importance of temperature for all aspects of insect life [29], the survival of intra-puparial forms inside hermetic containers will be under some influence of temperature conditions inside the container.

### Study limitations

#### No control over initial stages of development

We used larvae bought in a fishing shop and therefore had no control over the conditions of rearing at the initial stages of insect development. However, the survival recorded in this study in open containers was high, similar to the laboratory rearing in optimal conditions. Accordingly, specimens used in this study were a realistic representation of wild populations.

#### Developmental rate in the containers

The developmental rate in hermetic containers may be higher than that in optimal conditions [30]. Therefore, the time of intra-puparial development used to calculate the predicted development in the container might have inaccurately represented the times of development in airtight containers. Accordingly, air volumes per puparial weight per day, which were obtained during the experiments, may also be inaccurate (too low). However, at present, better models are out of our reach, as there are no scientific data on developmental rate of blowflies in hermetic conditions.

## Key points

1. The survival of intra-puparial forms in hermetic containers was studied for *Lucilia sericata* and *Calliphora vomitoria*.
2. For both species survival in such conditions was largely affected by the container volume, the number of puparia inside, and their age.
3. Below 0.05 ml of air per 1 mg of puparium per day of the development inside the container no insect survived. At or above 0.2 ml of air per 1 mg of puparium per day 100% of insects survived.
4. The study has indicated that blowflies reveal a single, general pattern of survival in hermetic conditions.
